# Air Pollutants in Puerto Rico: Key Pollutants and Carcinogenic Properties

**DOI:** 10.3390/ijerph22101549

**Published:** 2025-10-11

**Authors:** Devrim Kaya, Clara Santiago, Enrique Pernas, Sammy Truong, Greicha Martinez, Loyda B. Méndez, Yamixa Delgado

**Affiliations:** 1 School of Public Health, San Diego State University, San Diego, CA 92182, USA; 2 School of Naturopathic Medicine, Universidad Ana G. Mendez Gurabo Campus, Gurabo, PR 00777, USA; santiagoc7@uagm.edu; 3 Biochemistry & Pharmacology Department, San Juan Bautista School of Medicine, Caguas, PR 00726, USA; 4 Microbiology Program, Natural Science Department, Interamerican University Metropolitan Campus, San Juan, PR 00919, USA; 5 School of Science and Technology, Universidad Ana G. Mendez Carolina Campus, Carolina, PR 00983, USA; lbmendez@uagm.edu

**Keywords:** carcinogens, hazardous air pollutants (HAPs), health risks, industrial emissions, natural contaminants, Puerto Rico, cancer

## Abstract

Air pollutants pose a growing public health concern in Puerto Rico (PR), particularly from rapid industrialization, military activities, environmental changes and natural disasters. A total of 193 pollutants, comprising the 187 hazardous air pollutants and the 6 criteria air pollutants—including particulate matter (PM), carbon monoxide (CO), volatile organic compounds (VOC), and heavy metals—coincide with rising respiratory disease rates (e.g., lung cancer) documented in national and regional health registries. This study aimed to review major air pollutants in PR, their molecular carcinogenic mechanisms (mostly focused on respiratory-related cancers), and the geographic areas impacted significantly. We conducted an extensive literature search utilizing peer-reviewed scientific articles (PubMed and Web of Science), governmental reports (EPA, WHO, State of Global Air), public health registries, (Puerto Rico Central Cancer Registry and International Agency for Research on Cancer) and local reports. Data on pollutant type, source, molecular pathways, and carcinogenic properties were extracted and synthesized. Our analysis identified ethylene oxide (EtO), polycyclic aromatic hydrocarbons, and PM from industrial sites as key pollutants. The municipalities of Salinas and Vieques, hubs of industrial activity and military exercises, respectively, emerged as critical hotspots where high concentrations of monitored pollutants (e.g., EtO, formaldehyde, 2,3,7,8-tetrachlorodibenzo-p-dioxin (TCDD) and diesel PM) are associated with a significant prevalence of cancer and respiratory diseases. These agents, known to induce genomic instability and chromosomal aberrations, were correlated with elevated local cancer incidence. Our findings underscore the urgent need for targeted public health interventions and support a multi-pronged strategy that includes: (1) enhanced regulatory oversight of EtO and other hazardous air pollutant emissions; (2) community-based biomonitoring of high-risk populations; and (3) investment in public health infrastructure and a transition to cleaner energy sources. Integrating rigorous environmental science with public health advocacy is essential to strengthen PR’s cancer-control continuum and foster resilience in its most vulnerable communities.

## 1. Introduction

Air pollution is a major global environmental health risk for morbidity and mortality, contributing substantially to the burden of chronic diseases, including cancer. According to the State of Global Air, air pollution was associated to 8.1 million deaths worldwide in 2021 [1], ranking it as the second most significant risk factor for premature death globally [1]. Previously, the World Health Organization (WHO) indicated that ambient (outdoor) air pollution was associated to approximately 4.2 million premature deaths globally in 2019, with a significant proportion attributed to noncommunicable diseases such as lung cancer, cardiovascular diseases, and chronic respiratory conditions [2]. Outdoor air pollution, particularly particulate matter (PM)_2.5_, was officially classified as carcinogenic to humans (Group 1) by the International Agency for Research on Cancer (IARC) in 2013 [3], with sufficient evidence linking exposure to increased lung cancer risk and limited evidence for other types [4]. Emerging evidence suggests that air pollution may play a significant role in driving lung cancer in non-smokers, highlighting the need for stronger environmental health protections [5,6]. These findings highlight the critical importance of understanding the components of air pollution that contribute to its carcinogenicity.

Puerto Rico (PR) is an unincorporated territory of the United States located in the northeastern Caribbean, within the Greater Antilles. Although commonly referred to as an island, PR is actually an archipelago consisting of the main island and several smaller islands and islets, some inhabited (such as Vieques and Culebra) and others uninhabited. Its southern borders face the Caribbean Sea, while its northern and eastern shores face the Atlantic. The main island of PR is divided into three geographical regions: mountains, coastal lowlands, and karst country. This geographical makeup of PR combined with densely populated urban centers can exacerbates environmental health challenges In addition, its industrial history, military activities, and recent natural disasters (e.g., hurricanes) have contributed to a complex air pollution profile characterized by elevated levels of PM, volatile organic compounds (VOCs), heavy metals, and persistent organic pollutants (POPs) across the island [7,8,9,10]. Particularly, the destruction of PR’s electrical grid by Hurricane Maria in 2017 led to a surge in the use of backup diesel generators, markedly increasing combustion-derived emissions of PM, nitrogen oxides (NO_x_), carbon monoxide (CO), and hazardous air pollutants (HAPs) across the island [7]. Vieques, an island municipality of PR, experienced decades (from the 1940s until 2003) of US Navy bombing exercises that released unique toxicants such as depleted uranium (DU), heavy metals, and explosives residues, raising concerns over potential carcinogenic risks in exposed populations [11,12,13,14,15,16]. In addition to local sources, transboundary pollution events, such as Saharan dust intrusions, periodically introduce additional PM and associated metals to Puerto Rican air, further complicating the exposure landscape [17]. According to 2022 cancer statistics for Puerto Rico, lung cancer ranked as the fourth most diagnosed cancer for both men (5.4% of new cases) and women (4.0%) [18]. Despite this incidence ranking, lung cancer demonstrated a disproportionately high mortality burden, ranking as the second most lethal cancer for both sexes and accounting for 11.5% of cancer-related deaths in men and 8.9% in women [18]. This significant lethality of lung cancer directs attention toward potential environmental contributors, particularly in regions of PR with documented histories of high air pollution.

A study conducted in November 2017 [7] deployed four real-time affordable multi-pollutant (RAMP) monitors and a black carbon monitor in the San Juan Metro Area. During the first month of data collection, these monitors recorded high levels of CO and sulfur dioxide (SO_2_) every night. Observations indicated higher PM_10_ concentrations in the most densely populated areas, including San Juan, Bayamon, Guaynabo, Cataño, and Carolina [19]. These areas host major industrial sources, such as power plants, refineries, ports, an airport, landfills, dense traffic, and road dust resuspension [19].

In PR, up to 59 trace elements have been measured in airborne PM samples collected from various sites, which are categorized as either rural or urban [10]. Studies have shown that rural areas tend to have higher levels of certain metals compared to urban areas. Conversely, other metals, commonly associated with fossil fuel combustion and biomass burning, were found at elevated levels in urban areas. Additionally, trade winds occasionally bring in dust from distant sources. To facilitate the monitoring of HAPs, Sen-Severe, an institution in Pittsburgh, PA, collaborated with Carnegie Mellon University to develop RAMP monitors. These monitors have been utilized in various scientific investigations to quantify and analyze HAP emissions various regions in San Juan [7]. Although RAMPs are not federally recognized as equivalent methods, their data can meet US Environmental Protection Agency (EPA) quality standards for monitoring of nitrogen dioxide (NO_2_), ozone (O_3_) and CO [7].

Despite these challenges, air quality monitoring infrastructure in PR remains limited, with significant data gaps persisting in the characterization of HAPs and their health effects on the island’s population. Low-cost sensor networks and community-engaged research initiatives have attempted to address these gaps, yet comprehensive epidemiological and mechanistic studies linking exposure to specific pollutants with carcinogenic outcomes are still urgently needed [7].

This review examines major sources of HAPs, key pollutants, mechanisms of carcinogenesis-focusing on emissions from military, industrial, and natural sources. We highlight pollutants of concern, including PM, VOCs, NO_x_, CO, SO_2_, O_3_, heavy metals, POPs, ammonia (NH_3_), and military-specific contaminants like DU. By synthesizing findings from epidemiology, toxicology, and environmental monitoring, we aim to identify critical knowledge gaps and propose future research directions to better understand and mitigate air pollution-related cancer risks in PR.

## 2. Methodology

This manuscript presents a descriptive review of the literature. This approach was intentionally chosen to synthesize a broad and comprehensive overview of environmental contaminants and health risks in PR, a topic for which data is often dispersed across various scientific and governmental sources. This review summarizes findings from air quality monitoring, epidemiological studies, and risk assessment models to characterize air pollution in Puerto Rico. It is important to note that this review describes observed associations and modeled risks; it does not aim to establish direct causal relationships.

The literature search was conducted from January 2025 to July 2025 using the PubMed and Web of Science databases. To compile relevant gray literature, we performed targeted searches of official reports and databases from key agencies and organizations, including: Puerto Rico Central Cancer Registry, IARC, Puerto Rico Department of Health, the EPA—with a focus on compliance with standards like the National Ambient Air Quality Standards (NAAQS)—the National Institutes of Health (NIH), the National Aeronautics and Space Administration (NASA), the WHO, California’s Office of Environmental Health Hazard Assessment (OEHHA), the Health Effects Institute (HEI State of Global Air), and the non-profit organization Earthjustice.

Our search strategy employed various combinations of the following keywords: ‘Puerto Rico’, ‘Carcinogens’, ‘HAPs’, ‘Criteria Air Pollutants’, ‘Health Risks’, ‘Industrial Emissions’, ‘Natural Contaminants’, and ‘Cancer’. Articles and reports were selected based on the following criteria: Inclusion: (1) Peer-reviewed articles or official reports with a primary focus on environmental pollutants and/or associated health risks in Puerto Rico; (2) publications written in English or Spanish. Exclusion: (1) Studies not specific to the Puerto Rican context; (2) opinion pieces, personal blogs, or other non-scientific commentaries.

To ensure accuracy, we prioritized literature published within the last 10 years. However, foundational studies or topics with limited recent data were included with an expanded date range of up to 25 years to provide essential context. While the review is anchored in scientific and official literature, we also included a limited number of online news articles from reputable sources. These were not used as a source for primary data but were included sparingly to provide context for specific, localized environmental health events. This structured selection process resulted in a final corpus of 138 references that form the basis of this review.

For Table 1, we reported the maximum allowable concentration for each pollutant based on the NAAQS, and attainment status based on EPA and the Puerto Rico Department of Natural and Environmental Resources (DRNA). For Table 2, the carcinogenic properties were reported in accordance with the EPA classifications from the Integrated Risk Information System (IRIS) or the National Toxicology Program. For Table 3, the midpoint concentration was calculated as the arithmetic mean of the reported minimum and maximum concentration range. This value was used to calculate the representative Lifetime Cancer Risk (LCR) and Hazard Quotient (HQ). 

To provide a quantitative estimate of uncertainty, a sensitivity analysis was performed by calculating the LCR and HQ using both the minimum and maximum reported concentrations for each pollutant. The resulting range (LCR_min_ to LCR_max_ and HQ_min_ to HQ_max_) defines the potential exposure risk span. These full ranges are detailed in Supplementary Materials Table S1.

## 3. Overview of Air Pollutants: Definitions and Classifications

Anthropogenic sources that release pollutants and hazardous materials into the atmosphere have multiplied exponentially since the onset of the industrial revolution. Examples of anthropogenic sources include industrial emissions, vehicle exhaust, fossil fuel combustion, and military activities. This has gradually degraded air quality, especially in urban and industrialized regions.

The WHO defines air pollution as the presence of harmful pollutants, such as smoke, gases, or vapors, in the atmosphere at concentrations and lengths of time that may be harmful to human health. Air pollutants are typically categorized into two major groups: criteria pollutants and HAPs.

Criteria pollutants are six common air pollutants—PM_10_, PM_2.5_, ground-level O_3_, NO_2_, SO_2_, CO, and lead (Pb)—regulated under the US Clean Air Act due to their widespread presence and well-documented health impacts [20]. They are spread across the country and can harm public health and the environment. These pollutants have strong evidence linking them to adverse health outcomes [21]. The EPA has established the NAAQS, maximum allowable concentration for each pollutant. Table 1 summarizes their major sources and health impacts.

**Table 1:** Criteria air pollutants, standards, and health Effects [4,21,22,23,24,25,26].

Pollutant	NAAQS	Current Status in Puerto Rico	Major Sources	Key Health Effects
PM_2_._5_	Primary Annual: 12 µg/m^3^ Primary 24 h: 35 µg/m^3^	In attainment. However, significant exceedances occur during Saharan dust events.	Combustion: Vehicles Power plants Fires Natural: Construction dust Saharan dust	Lung irritation and cancer Asthma Heart attack Group 1 carcinogen.
PM_10_	Primary 24 h: 150 µg/m^3^	In attainment		Respiratory irritation Aggravation of respiratory conditions like asthma and bronchitis Group 1 carcinogen
O_3_	Primary 8 h: 0.070 ppm	In attainment	Not directly emitted Formed from NOx + VOCs in the presence of sunlight.	Coughing and throat irritation Asthma attacks Impaired lung development in children Oxidative stress on the respiratory tract
NO_2_	Primary 1 h: 100 ppb Primary Annual: 53 ppb	In attainment	Combustion: Vehicle exhaust Power plants Contributes to smog and secondary PM formation	Airway inflammation Asthma aggravation Increases susceptibility to infections
SO_2_	Primary 1 h: 75 ppb	In attainment	Combustion: Power plants Ships Other coal and oil combustion sources	Bronchoconstriction Wheezing Shortness of breath
CO	Primary 1 h: 35 ppm Primary 8 h: 9 ppm	In attainment	Incomplete combustion: Vehicles Generators Fires	Reduces oxygen delivery to tissues (forms carboxyhemoglobin) Headaches and dizziness at moderate levels Fatal at high levels.
Pb	Primary 3-month average: 0.15 µg/m^3^	In attainment	Historically, leaded gasoline Metal smelters Battery recycling Old paint dust	Neurotoxin, cognitive impairment and behavioral problems in children Hypertension in adults Kidney damage in adults Probable carcinogen.

Note: These are the six “criteria” pollutants regulated by NAAQS. While not the focus of this review, they form the backdrop of general air quality in PR and can indirectly indicate the presence of co-emitted toxic pollutants. Current attainment status is based on the most recent data from the EPA and the Puerto Rico DRNA. Standards are subject to periodic review by the EPA.

PR generally complies with NAAQS for most criteria pollutants; however, in 2023, the EPA designated San Juan and Guayama-Salinas as non-attainment areas for SO_2_, largely due to emissions from power plants [27]. In 2024, emission reduction plans targeting PREPA’s San Juan, Palo Seco, and Aguirre facilities were approved [27].

HAPs, also known as air toxics, comprise 187 (excluding methyl ethyl ketone, 2005, and caprolactam, 1996) chemicals identified in the US Clean Air Act Amendments of 1990 for their potential to promote cancer and other serious health effects [28]. These include VOCs like benzene and formaldehyde, heavy metals like arsenic and mercury, POPs like dioxins polycyclic aromatic hydrocarbons (PAHs), industrial byproducts, pesticides, and military-specific toxics like DU [29]. HAPs are less widespread than criteria pollutants but can be highly toxic.

Unlike criteria pollutants, HAPs are not regulated through NAAQS but through technology-based emissions standards under the National Emissions Standards for Hazardous Air Pollutants (NESHAP). Even at low concentrations, long-term exposure to HAPs can significantly increase the risk of cancers (e.g., leukemia from benzene exposure) and a myriad of non-cancer outcomes, including birth defects, and neurological disorders [29]. Notably, many HAPs, such as mercury and persistent organics, can also contribute to local or regional environmental problems through deposition onto soil and water, subsequently bioaccumulating in food chains and creating indirect exposure routes.

PR follows the US EPA regulations, implementing emission limits such as Maximum Achievable Control Technology (MACT) standards for HAPs. Among these pollutants, only lead is subject to NAAQS. In contrast, benzene, arsenic, chromium VI, EtO, and PM containing carcinogens are regulated primarily through emissions control technologies rather than ambient air limits.

To better understand pollutant exposure, the EPA developed the Air Quality System (AQS) database, which offers amounts of air pollutants measured through thousands of monitoring stations across the US, including PR [30]. This resource allows researchers to assess pollutant levels historically (1980–2024) and in real-time, supporting studies of daily, seasonal, and long-term exposures [30].

In PR, concerns about HAP exposures have intensified over the past decade due to multiple high-profile events and findings: the identification of extreme cancer risk “hotspots” near industrial facilities [31], controversies over coal ash disposal in the south of the island [32], and the aftermath of natural disasters like Hurricane María (2017) which led to prolonged island-wide generator use and debris burning [7]. As a result, PR’s residents suffer elevated rates of asthma, cancer, diabetes, and heart disease in certain areas, raising questions about the contribution of environmental pollutants [33].

## 4. Sources of HAPs in PR

Scientific research has focused on a number of industrial sites in PR because of the significant increases in the prevalence of various cancers and other diseases that pose a threat to the general health of those who live in or near these areas [9,10].

**Figure 1:**
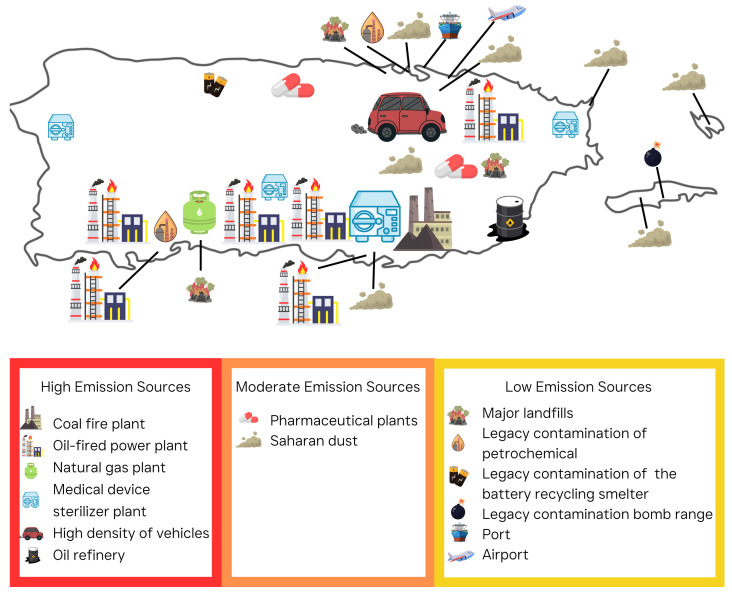
Geographic distribution and qualitative contribution of major air pollution sources across (PR). This schematic aims to visually communicate relative air pollution burdens across the island, grounded in measured or modeled concentrations and risk metrics. The figure was generated based on midpoint air pollutant concentrations (μg/m^3^) reported in Table 2, using available monitoring data from 2015 to 2023. Icons are scaled proportionally to reflect the relative magnitude of emission, with larger icons representing higher estimated regional contributions. These include power generation facilities (coal, oil, and natural gas), medical device sterilizer plants emitting HAPs like EtO, and pharmaceutical manufacturing centers [9,19,27,31,32]. The San Juan metropolitan area is marked by high vehicle density, port, and airport activity, contributing to air pollution [10,19]. The south region (Guayama–Peñuelas) continues to host the island’s largest fossil fuel infrastructure. Legacy contamination sites—such as a petrochemical complex in the south, a former battery smelter in the north, and a former military bombing range in Vieques—pose ongoing environmental health risks [8,9,10,11,16,34,35]. Poorly managed major landfills also contribute various HAPs.

As a result, PR’s industrial history and energy infrastructure have created a mosaic of HAP emission sources across the island. Figure 1 highlights several major pollution hotspots in PR, reflecting clusters of industrial facilities and other sources of air toxics; several of these locations have been designated as Superfund sites by the US EPA due to historical contamination. Broadly, sources of HAPs in PR include both stationary sources (such as industrial or commercial facilities, power generation, and waste sites) and mobile sources (including transportation). Figure 1 maps major HAP emission hotspots across PR, including fossil fuel plants, EtO sterilizer sites, transportation hubs, and legacy military zones.

*Stationary Combustion Sources and the Energy Sector:* The island’s electrical grid and, thus, the island’s local air monitoring network suffered significant setbacks during Hurricane Maria in 2017. As a result, power outages have become more frequent and many residents relied on these generators for extended periods (primary combustion sources) [7], introducing large quantities of combustion-related pollutants such as CO, SO_2_, black carbon, and VOCs into the air [7]. These emissions included hazardous air toxics like benzene and formaldehyde.

Electric power in PR has traditionally relied on fossil fuels, including coal and oil. The Applied Energy Services (AES) Puerto Rico coal-fired power plant in Guayama (southeast PR) began operation in 2002 under a 25-year contract with Puerto Rico Electric Power Authority (PREPA) and has since been a significant source of air toxics and community concern [32]. This plant emits not only criteria pollutants (SO_2_, NO_x_, PM) but also mercury, arsenic, chromium, nickel, cobalt, radium and other metals found in coal, as well as acid gases and organic HAPs from combustion. Notably, AES Puerto Rico generates over 300,000 tons of coal ash annually from its Guayama facility. AES’s operations have left a legacy of coal ash, the residual waste, stored in a large uncovered pile on-site and previously distributed as fill material off-site [32,36]. Indeed, an epidemiological study by the University of Puerto Rico Graduate School of Public Health found higher rates of respiratory and cardiovascular diseases, asthma, skin rashes, and even spontaneous abortions in the Guayama community compared to a control area, potentially associated to chronic exposure to AES’s emissions and ash [32].

Besides AES, PR has oil-fired power plants (e.g., at Palo Seco west of San Juan, Aguirre in Salinas, and Costa Sur in Guayanilla/Ponce). These plants emit SO_2_ and nickel compounds (from residual oil) and contributed to past NAAQS violations [27]; they likely also emit HAPs such as VOCs and PAHs from incomplete combustion. In recent years, natural gas has been introduced (e.g., EcoElectrica LNG plant in Penuelas), which can reduce some emissions but still produces formaldehyde and other HAPs from gas turbines.

*Industrial and Manufacturing Emissions:* During the mid-20th century, particularly in the aftermath of World War II, the Puerto Rican government implemented a comprehensive economic initiative aimed, known as “Operation Bootstrap” (*Operación Manos a la Obra* in Spanish). The objective was to transition PR from an agricultural to an industrialized economy. The initiative promoted the investment of factories from US and foreign countries in the areas of textiles and petrochemicals from 1947 to 1970, then, pharmaceuticals, medical devices and technology from 1970 to 2000. Consequently, the manufacturing sector in PR is another major HAP source.

One contemporary issue has been emissions of EtO from commercial sterilization facilities. PR, surprisingly, is home to seven medical device sterilizer plants (reflecting the large medical device industry on the island), the highest concentration of such facilities relative to population in the US [31]. These facilities use EtO gas, a potent human carcinogen, to sterilize medical equipment, which can leak or be vented to the atmosphere. In 1998, the EPA identified four PR sterilizers (in Salinas, Añasco, Fajardo, Villalba) as among the most hazardous in the nation with extremely high EtO concentrations (>121 µg/m^3^) and with modeled cancer risks well above 100 per million for nearby residents [31]. In fact, the Steri-Tech EtO plant in Salinas has been reported to confer an excess cancer risk on the order of 6000 per million for those closest to it [31], an astonishingly high risk driven almost entirely by EtO emissions. These facilities also garnered attention due to regulatory violations: for example, Steri-Tech (Salinas) was cited by Occupational Safety and Health Administration (OSHA) in 2019 and EPA in 2021 for improper handling and under-reporting of EtO emissions, and faced fines for exposing workers and communities to the carcinogen [37].

Other industrial sources in PR include petroleum handling and chemical storage (e.g., fuel terminals to supply transport and generators), which release hydrocarbon and VOC emissions (like benzene and toluene) especially if there are leaks or during tank loading. Commonwealth Oil Refining Company (CORCO) and Caribbean Petroleum Corporation (CAPECO), a major oil refinery and storage complex in Peñuelas/Guayanilla and Cataño/Bayamón, operated in past decades and likely contributed significantly to these emissions [38] and soot deposition in nearby communities [34]. In the 2000s, the EPA and Puerto Rico Environmental Quality Board investigated the emissions of lead and arsenic into the surrounding environment from the Battery Recycling Company in Arecibo [39].

Waste management sites represent another significant source of HAPs in PR. Many of the island’s 29 landfills were constructed in the 1950s and 1960s without modern gas emission controls, resulting in the uncontrolled release of landfill gases. These emissions include hazardous pollutants like VOCs, heavy metals, methane and NH_3_. Furthermore, past proposals for waste-to-energy incinerators, like the controversial project in Arecibo of the Energy Answers International (2012–2017), have raised serious public health concerns regarding potential emissions of highly toxic compounds, including dioxins and mercury [40].

PM_2.5_ air quality in PR from industrial sources is currently limited due to insufficient monitoring and research. Available information lacks the necessary detail on the measurement, sources and potential health impacts of PM_2.5_ [9]. Although a few studies have assessed individual chemicals from industrial sources, industrial facilities are recognized as sources of carcinogenic air pollutants [24,25,32,34,36,37,39,41]. These include NO_2_, SO_2_, CO, PM, VOCs, EtO, formaldehyde, dioxin, PAHs, benzene, 1,3-butadiene, and heavy metals such as lead, arsenic, mercury, chromium, cobalt, lithium, molybdenum, radium, selenium. Populations living near these sites in PR experience unequal exposure burdens [39]. Although all regions in PR are now in attainment for criteria pollutants, the industrial sources responsible for historical emissions remain a focus for air quality assessment.

*Mobile Sources and Transportation:* With over 3 million residents and heavy tourism, PR has a high density of vehicles, especially in the San Juan metropolitan area. Monitoring for criteria pollutants in high-density traffic corridors serves as an important indicator for HAP exposure. The same vehicle emissions that produce criteria pollutants like NO_2_ and PM_2.5_ are also major sources of a mixture of carcinogenic HAPs. Vehicle exhaust is a significant contributor to urban air toxics: gasoline-powered cars and trucks emit benzene, 1,3-butadiene, acetaldehyde, and formaldehyde (all carcinogenic or toxic), while diesel engines (buses, trucks, backup generators) release a particularly harmful mix of PM with adsorbed organics (diesel PM is classified by IARC as carcinogenic to humans, largely due to lung cancer risk) [41,42].

The northern coast of PR, particularly San Juan and surrounding municipalities, exhibits higher PM_2.5_ levels due to dense urbanization and industrial activity [10]. In contrast, southern regions contribute significantly to air pollution through stationary sources, including multiple power plants that have operated for decades [9,43].

Additionally, ports and airports represent high-emission zones, resulting in significant residents’ exposure to mobile-source HAPs. The Port of San Juan, one of the busiest in the Caribbean, is a significant source of PM, sulfur compounds, and PAHs due to emissions from idling diesel ships and cargo vessels. Similarly, airline traffic through San Juan’s airport, Luis Muñoz Marín International Airport, contributes ultrafine particles and VOCs.

Following the collapse of PR’s electrical grid during Hurricane María (2017), widespread reliance on backup diesel generators created acute, hybrid stationary/mobile pollution sources [7]. Air monitoring data from the post-María period revealed sharp increases in SO_2_, CO, and black carbon, attributed to intense generator usage [7]. These generators emitted additional HAPs such as benzene and formaldehyde, compounding the respiratory and cardiovascular risks during disaster recovery periods. This implies that disaster scenarios can acutely increase mobile/stationary hybrid sources.

*Natural and Episodic Sources:* The link between criteria pollutants and HAPs is evident in natural phenomena affecting the island. PR is seasonally affected by Saharan dust transported across the Atlantic each spring and summer [44]. These natural events are known to promote exceedances of the PM_2.5_ air quality standard, also transport hazardous components such as carcinogenic crystalline silica and potentially adsorbed pesticide residues. Metro area (San Juan, Guaynabo and Cataño) and eastern municipalities (Fajardo, Vieques and Culebra) are the first impacted due to easterly trade winds [45]. Southern areas such as Salinas, Ponce and Guayama often report higher concentrations due to drier conditions and wind channels [46]. During peak events (June–August), central regions like Caguas still experience worsened air quality, particularly impacting children, the elderly, and individuals with respiratory conditions [46]. These plumes contain PM_2.5–10_, rich in silica, trace metals, and sometimes microbial contaminants, and have been associated with increased respiratory irritation, asthma exacerbations, and emergency room visits [17,47]. Although mineral dust is not listed as a HAP, its particulate composition and ability to adsorb other toxics make it a significant episodic source of airborne health risk (e.g., asthma exacerbations) [17]. Occasionally, volcanic emissions from Caribbean sources (e.g., Montserrat’s Soufrière Hills) reach PR, contributing sulfurous haze. Though infrequent, these events temporarily degrade air quality [48].

*Agricultural and Open Burning Sources*: While large-scale sugarcane field burning has declined with agricultural shifts, open burning of household waste and storm debris remains common, particularly in rural areas with limited waste management infrastructure [10,19,49]. This practice emits a complex mixture of HAPs including dioxins/furans, benzene, styrene, and PAHs from the combustion of plastics and other materials [50]. After Hurricanes Irma and María in 2017, illegal or informal burning of debris was reported, which raised concerns over PAHs and dioxins in affected communities [51]. Despite known health risks, such burning remains largely unregulated and constitutes a substantial localized hazard. Moreover, pesticide use in agriculture poses airborne risks through volatilization. Compounds such as chlorpyrifos, paraquat, and atrazine may drift into ambient air, though empirical data for PR remains limited [52,53,54].

*Biogenic and Soil Emissions*: PR’s natural landscapes contribute to air pollution via biogenic volatile organic compounds and soil emissions of NO_x_. Emissions of isoprene, monoterpenes, and n-alkanes from vegetation, along with n-alkanoic acids from biomass decomposition, are well-documented precursors to secondary organic aerosol (SOA) formation, especially during the summer due to enhanced photochemical activity [55,56,57,58]. Soils account for approximately 15% of global NO_x_ emissions, influenced by microbial activity, moisture content, and fertilizer application [59]. These emissions, while natural, interact with anthropogenic pollutants to elevate O_3_ and aerosol levels, contributing around 75–90% of annual global SOA production [60].

In addition, comprehensive air sampling across PR has identified region-specific patterns: alkanes, esters, phthalates, and siloxanes dominate in the south; phenyl phosphates and n-alkanoic acids are prevalent in urbanized areas like Bayamón and Humacao [9].

Table 2 summarizes major sources of HAPs in PR, ranging from energy and manufacturing sectors to legacy military contamination and natural dust events. Across these source categories, a clear pattern of environmental injustice emerges: communities located near high-emission facilities, such as the AES coal plant in Guayama and EtO sterilizers in Salinas, Añasco, and Fajardo, are often low-income, predominantly Afro-Puerto Rican, and face disproportionate exposure burdens [31,32]. These patterns highlight the need for targeted, community-based monitoring strategies and more stringent regulatory controls to mitigate the public health risks associated with HAPs.

**Table 2:** Major sources of hazardous air pollutants (HAPs) in Puerto Rico (PR).

Source Category	Potential Sources in PR	Key Carcinogenic Pollutants Emitted	Carcinogenic Classification Summary (IARC/EPA) [61,62]
Coal-Fired Power Plant	AES-PR Guayama coal plant	Arsenic, Chromium VI, Nickel, PAHs, Lead	Known/Group 1 (Arsenic, Chromium VI)
Oil-Fired Power Plants	PREPA plants (multiple locations)	Benzene, Formaldehyde, Nickel, Vanadium	Known/Group 1 (Benzene, Formaldehyde)
Medical Device Sterilizers	Steri-Tech (Salinas), Edwards, Lifesciences Technology (Añasco), Customed (Fajardo), and Medtronic (Villalba)	Ethylene Oxide (EtO)	Known/Group 1
Pharmaceutical and Chemical Mfg.	Pharma plants in Barceloneta	Methylene chloride, Chloroform	Probable/Group 2A (Methylene chloride)
Petrochemical Storage	Peñuelas and Yabucoa oil terminals	Benzene, Vinyl chloride, Styrene	Known/Group 1 (Benzene, Vinyl chloride)
Waste Disposal	Landfills (multiple), Open Burning	Dioxins, PCBs, Benzene, Mercury	Known/Group 1 (Dioxins, PCBs)
Transportation–Road	~3 million vehicles	Benzene, 1,3-butadiene, Diesel PM	Known/Group 1
Transportation–Marine/Air	Port of San Juan, SJU Airport	Benzene, PAHs, Formaldehyde, Nickel	Known/Group 1 (Benzene, PAHs)
Natural Dust Events	Saharan dust episodes	Crystalline Silica (in PM)	Known/Group 1
Military/Ordnance	Vieques bombing range (historic)	RDX, 2,4,6-trinitrotoluene (TNT), Lead, Uranium	Probable/Group 2A (TNT)
Agricultural Emissions	Sugar cane burning, Fumigation	Benzo[a]pyrene (BaP), Atrazine, Paraquat	Known/Group 1 (BaP), Group 2B (Atrazine)

Note: The above list is not exhaustive of all HAPs but focuses on those relevant to PR’s context. Group 1 = carcinogenic to humans; 2A = probably carcinogenic; 2B = possibly carcinogenic (IARC). EPA classifications are based on IRIS or the National Toxicology Program: many listed “known” or “likely” carcinogens. All listed pollutants also have significant non-cancer health effects (e.g., neurotoxicity for lead, immunotoxicity for dioxins, etc.).

## 5. Carcinogenic Pollutants and Toxicological Evidence in PR

A consolidated overview of the island’s major HAP sources, associated pollutants, and classifications by IARC and the US EPA’s IRIS is presented in Table 2 [63]. Among these HAPs present in PR’s atmosphere, a subset stands out as particularly important due to their prevalence, toxicity, and associations with local sources. Many of these pollutants are known human carcinogens (Group 1 IARC; “known” or “likely” human carcinogen per EPA), and they are also associated with an increased risk of other health disorders (e.g., neurological damage or respiratory disease). This section focuses on pollutants that either: (1) dominate the estimated cancer risk in PR based on exposure assessments, or (2) have been the subject of public and scientific concern due to their prevalence or regulatory attention. Additionally, despite growing concerns about environmental health disparities, research directly linking HAP exposure to health outcomes in PR remains limited. However, a synthesis of available evidence from modeled risk assessments, environmental monitoring, epidemiological studies, and community health surveys suggests significant public health implications, particularly regarding carcinogenic risk. According to the US EPA’s National Air Toxics Assessment and the updated AirToxScreen, several Puerto Rican census tracts, including Guayama, Salinas, and San Juan Metro, show elevated estimated cancer risks, primarily driven by ethylene oxide, formaldehyde, and benzene [64].

*Benzene and 1,3-Butadiene:* Both benzene and 1,3-butadiene are emitted from vehicle exhaust, industrial activities, and open burning. Benzene is a well-established human carcinogen (IARC Group 1), associated with leukemia and other hematologic malignancies. 1,3-butadiene, also Group 1, increases risks of lymphomas and leukemias [63,65]. In PR’s urban centers, especially along high-traffic corridors, these pollutants are among the most significant contributors to air toxic-related cancer risk. They are relatively reactive in air, so concentrations can vary over short distances, at the highest near busy roads or industrial sources like refineries.

*Formaldehyde and Acetaldehyde:* These carbonyl compounds, formaldehyde (Group 1) and acetaldehyde (Group 2B) [63], are formed both directly through combustion (e.g., vehicle engines, power plants) and secondarily via atmospheric photochemical reactions from other VOCs. Formaldehyde is correlated to nasopharyngeal cancer and possibly leukemia [63,65]. Warm, sunny conditions in PR favor secondary formation, leading to elevated background concentrations.

*Ethylene Oxide (EtO):* EtO is a potent alkylating agent used in commercial sterilization. It is classified as a Group 1 carcinogen and is associated to lymphoid and breast cancers [63], and in 2016 the EPA’s IRIS assessment found EtO to be far more carcinogenic than previously thought, with an inhalation unit risk that makes it one of the most hazardous air toxics on a per-unit basis [66]. According to the EPA 2014 National Air Toxics Assessment (NATA), several communities in PR face a significantly elevated LCR due to EtO emissions from four medical sterilization facilities: Steri-Tech in Salinas, Edwards Lifesciences Technology in Añasco, Customed in Fajardo, and Medtronic Puerto Rico Operations Co. in Villalba [67,68,69,70]. The NATA analysis, which utilized the AERMOD dispersion model, estimated these risks at the census tract level. For the Steri-Tech, Inc. in Salinas, the model estimated a maximum risk of 6000 in a million, one of the highest in the nation. Other high-risk areas included the tracts containing Edwards Lifesciences in Añasco (5000 in a million), Customed, Inc. in Fajardo (1000 in a million), and Medtronic PR Operation Co. in Villalba (800 in a million). While EPA’s risk threshold for EtO is 1–100 per million. Based on these EPA reports, in communities near PR’s sterilizer plants, EtO accounts for 90–96% of air toxics risk in the most impacted census tracts. For example, Añasco and Salinas tracts, adjacent to commercial sterilizers, rank among the highest nationwide for ambient HAP-related cancer risk. These estimates place affected communities among the most at-risk nationwide [37]. These values are dramatically above the typical US background air toxics risk (~30 per million) [64]. In Salinas, residents have reported cancer clusters near the EtO-emitting Steri-Tech facility, compounded by operational incidents such as explosions. Furthermore, OSHA citations of excessive EtO exposure among Steri-Tech workers has also indicated the urgent need for improved workplace protections and suggest potential environmental releases [37]. The presence of seven EtO-emitting plants on a small island is thus a major concern.

From a regulatory perspective, VOCs, including EtO, have been historically under-monitored, but recent EPA Region 2 deployments confirmed EtO presence near sterilizer facilities, although comprehensive datasets are still pending. Just recently in 2023, EPA moved to tighten EtO emission standards by requiring 99.99% emission reduction–a response that came after years of advocacy, including in PR where communities demanded action as early as 2016 [31,37]. Before these changes, companies were allowed to emit significantly more, explaining the high ambient risks observed. Notably, industrial sites in permanently inhabited regions of PR have reported releases of several carcinogenic chemicals, such as EtO (580 pounds) and benzene (6594 pounds) [39]. EtO’s health risk and recent enforcement in PR exemplify how emerging science can identify previously under-appreciated pollution problems.

*Polycyclic Aromatic Hydrocarbons (PAHs)*: PAHs are a class of organic chemicals formed during incomplete combustion of carbon-containing material (coal, oil, wood, tobacco, etc.). They include compounds like BaP, benzo[k]fluoranthene, and dozens of others. PAHs often adsorb onto soot particles. BaP is a Group 1 carcinogen (associated to lung and skin cancers via DNA-adduct formation) [4,63]. PAHs as a mixture are typically considered probable carcinogens. In PR, PAHs emanate from vehicle exhaust (especially diesel), open burning of waste or biomass, and the coal plant emissions. People in communities like Guayama (near the coal plant) or those exposed to a lot of vehicle smoke (e.g., busy intersections in San Juan) inhale PAHs. Aside from cancer, PAHs are known risk factors for respiratory and cardiovascular toxicity and even developmental effects if exposure occurs in utero [63]. Monitoring of specific PAHs in PR is limited, but deposition on soils near highways or around the AES plant has shown PAH contamination, suggesting significant emission sources.

*Metals (Arsenic, Chromium (VI), Nickel, Cadmium, Lead):* Several toxic metals are present in PR’s air from industrial sources, especially in coal ash-producing industries. Arsenic is found in coal (and thus in coal plant emissions and ash) and in residual oil; it is a Group 1 carcinogen, causing lung, skin, and bladder cancers upon chronic exposure via inhalation or drinking water [4,63]. Arsenic in airborne particulate form can be inhaled or ingested after settling [4]. Chromium (VI), used in some industrial processes and present in coal ash, is another Group 1 carcinogen (lung cancer via inhalation of chromate dust) [4,63]. Nickel compounds (from oil combustion or metal plating industries) are carcinogenic to the lung and nasal cavity (Group 1, e.g., nickel refinery dust) [4,63]. PR’s oil plants historically emitted nickel; monitoring around Palo Seco power plant in the early 2000s showed fine particles enriched in nickel and vanadium. Cadmium, emitted from waste burning and previously from battery recycling, is a probable carcinogen and a known kidney toxin [4]. Lead, while much reduced in air since leaded gasoline was banned, can still be emitted from smelters or legacy contaminated dust [4]. Lead is classified as probably carcinogenic (Group 2A by IARC) and definitively toxic to neurological development [63]. The Arecibo battery recycling facility (before closure) led to elevated airborne lead; some contaminated soil can still resuspend into air. However, lead levels were reported below NAAQS levels after the facility’s closure [36]. Beyond these site-specific cases, emerging research underscores that heavy metals such as cadmium, lead, and mercury released as HAPs by industrial activity can deposit in soil and water [71]. These pollutants can accumulate in ecosystems and enter the human body through multiple exposure routes, contributing to long-term health risks including carcinogenesis through mechanisms involving oxidative damage, inflammation, and interference with cellular processes [71].

*Dioxins and Furans:* These are POPs formed during combustion of chlorine-containing materials (e.g., burning plastic or medical waste, or as a byproduct of certain chemical processes). The most toxic dioxin TCDD is Group 1 carcinogen and is infamous from Agent Orange and Seveso. Dioxins is correlated to a range of cancers and other effects (e.g., endocrine disruption and immune dysfunction) [63]. In PR, dioxins could be released from any incineration of chlorine-containing materials. This includes open burning of trash (e.g., many household wastes like PVC plastic release dioxins when burned), and historically, a US Navy facility in Penuelas incinerated hazardous waste (including possibly Agent Orange) in the 1970s. Soil tests in some locations (e.g., Vieques) have found dioxin-like compounds. While routine ambient dioxin data are lacking, this pollutant class is worth noting due to its potency.

*Diesel PM:* Although not a single chemical, diesel exhaust particulate is often treated as a distinct air toxic in risk assessments. IARC classified diesel engine exhaust as carcinogenic to humans (Group 1) in 2012, based on sufficient evidence for lung cancer. In PR, diesel engines are prevalent in trucks, ferries, generators, and heavy equipment. Diesel PM is essentially a carbon core with a cocktail of HAPs on it (including PAHs, nitro-PAHs, and metals). Annual PM_2.5_ levels typically range between 6 and 10 µg/m^3^, below the 2005 WHO limit but exceeding the 2021 annual average guideline of 5 µg/m^3^ [72]. Episodic PM_10_ spikes during Saharan dust events can exceed 150 µg/m^3^ [46]. Chemical speciation indicates contributions from traffic emissions, sea salt, crustal dust, and power generation. Limited studies have also documented the presence of PAHs in PM_2.5_ samples from urban and rural areas. Additionally, San Juan and Guayama-Salinas were designated SO_2_ nonattainment zones in the 2010s due to emissions from the AES coal plant and Aguirre oil facility [27]. Elevated SO_2_ likely co-occurs with acid gases and metal-rich particulates, suggesting broader HAP exposure. Following Hurricane María, widespread generator use contributed to spikes in SO_2_ and black carbon, coinciding with increased respiratory hospitalizations [7]. Epidemiologically, long-term exposure to traffic-related PM (of which diesel is a key component) is associated to a higher risk of lung cancer [63]. PR also reports one of the highest asthma prevalence rates in the US [46]. In Cataño, increased asthma attacks were associated with elevated sulfur and PM levels [34]. In PR, diesel emissions from trucks, ferries, generators, and backup power systems contribute significantly to fine PM exposures and associated lung cancer risk.

*Coal Ash*: As a byproduct of coal combustion, coal ash is a major source of airborne and fugitive emissions in communities near coal-fired power plants such as the AES facility in Guayama. Coal ash continues contaminating air, soil, and groundwater on-site and off-site. Fugitive dust from the coal ash (often called “Agremax”) is a manufactured aggregate composed of a mixture of fly ash and bottom ash that are moistened with water to produce a solidified material. PR’s Law 40-2017 prohibits disposal of loose fly ash or bottom ash but does not ban the use of Agremax, considered as a solid waste. Between 2004 and 2012, over 2 million tons of Agremax were transported to the municipalities of Salinas, Humacao, Peñuelas, Ponce, Santa Isabel, Coamo, Caguas, Juncos, San Juan, Dorado, Arroyo, Guayama, Mayaguez, and Toa Baja often without appropriate containment [73], exposing communities to fugitive dust and raising environmental concerns. Communities downwind in Guayama, Humacao and Peñuelas have complained of coal ash dust blowing into homes and schools and fear increased cancers and other illnesses [36]. Community sampling in Guayama identified arsenic-rich coal ash transported via wind, prompting EPA enforcement against the AES plant for repeated opacity violations in 2021 [32]. Indeed, an epidemiological study from 2016 by the University of Puerto Rico Graduate School of Public Health found higher rates of respiratory and cardiovascular diseases, asthma, skin rashes, and even spontaneous abortions in the Guayama community compared to a control area, potentially associated to chronic exposure to AES’s emissions and ash [32]. While causal inference is limited, observed conditions are consistent with toxic exposures to coal ash constituents such as arsenic and PAHs. Independent analyses and EPA monitoring have shown that coal ash leaches toxic elements, such as arsenic, hexavalent chromium, cadmium, selenium, nickel molybdenum, and lithium, at concentrations exceeding federal thresholds, especially into the South Coast Aquifer, which serves approximately 140,000 people [74]. While coal ash as a whole is not classified by IARC, many of these leaches toxic elements are Group 1 carcinogens, and others like lead and crystalline silica are Group 2A, meaning exposure to coal ash dust or leachate carries significant carcinogenic risk [61]. Coal ash dust can also become airborne, contributing to inhalation exposures to these toxic metals [75]. Despite Puerto Rican law banning coal ash disposal in 2017, loopholes permitted continued use and storage of Agremax [76]. Monitoring at the AES site since 2018 has revealed repeated groundwater exceedances for carcinogenic and toxic metals, prompting a 2024 EPA enforcement agreement requiring AES to enhance monitoring, notify the public, and implement corrective action [77,78]. Inhalation or ingestion of ash-derived pollutants has been associated to increased risks of cancer, kidney and cardiovascular disease, and developmental harm, especially in low-income communities that disproportionately bear the burden of environmental contamination [32,71,79,80].

*Other Carcinogenic Pollutants:* Other notable HAPs in PR include: vinyl chloride (Group 1, is a known risk factor of a rare liver cancer; used in plastics industry-not currently produced in PR but could be present near PVC usage or waste) [28,61]; methylene chloride (solvent, Group 2A, used in manufacturing) [28,29,81]; chloroprene (used to make neoprene; not produced in PR, but worth noting as similar facilities exist in US causing local cancer risks) [28]; Polychlorinated biphenyls (PCBs) (legacy pollutants that volatilize from contaminated sites or old electrical equipment, probable human carcinogens) [28,29,61]; atrazine and paraquat (pesticides, Group 2B, with limited airborne exposure data but known toxicity when volatilized) [52,53]; Methyl tert-butyl ether (MTBE) and chloroprene (Group 2B, present in fuel additives and industrial processes elsewhere; relevant due to similar industry types in PR) [28,29,61]. Understanding the nature of these pollutants is crucial, but equally important is how they could harm.

Table 3 provides a detailed assessment of ambient air pollutants, including HAPs, concentrations across PR from 2000 to 2023, with a focus on urban, industrial, and agriculturally impacted zones. It integrates toxicological benchmarks, including IURs, RfCs, and derived metrics like LCR, CRL, and HQ.

Several pollutants, including EtO, formaldehyde, and diesel PM, exceeded conservative cancer or non-cancer risk thresholds, particularly in urban and post-hurricane contexts. Notably, acrolein and EtO present non-cancer HQ above 1, suggesting acute or chronic respiratory risks. The high cancer risk associated with TCDD (dioxin) and BaP reaffirms long-standing concerns over legacy and combustion-derived pollutants.

The majority of contaminants remain within regulatory thresholds; however, cumulative exposures and multi-pollutant synergies, particularly in underserved communities, underscore the importance of continued monitoring, epidemiological tracking, and source mitigation. This analysis reveals Salinas as a critical hotspot, being the municipality, most affected by a high burden of air pollutants.

In Puerto Rico, air pollutant levels reveal a complex risk profile that both aligns with and diverges from exposure patterns observed on the mainland US and neighboring Caribbean islands. For example, the mean benzene level in Salinas (~1 µg/m^3^) is nearly double the most recent US national average, which was 0.56 µg/m^3^ across 117 monitoring sites in 2023 [82]. Similarly, according to the WHO, outdoor background benzene concentrations in rural and suburban/urban areas globally range from 0.99 to 2.3 µg/m^3^ [83], respectively, further contextualizing PR’s midrange values. In contrast, EtO levels in Salinas are strikingly elevated, with a midpoint of 60 µg/m^3^, which far exceeds typical ambient background concentrations in the continental US (0.136 μg/m^3^ to 0.407 μg/m^3^) in samples taken from October 2018 to September 2019 from 18 National Air Toxics Trend Stations and Urban Air Toxics Monitoring Networks [84]. However, particulate pollution trends present differently. PM_2.5_ levels in Ponce, Bayamón, and Humacao average 4.3–5.8 µg/m^3^. These figures are notably below the 2023 US national level mean of 8.2 µg/m^3^ [85]. Likewise, the PR’s neighboring island of the Dominican Republic had PM_2.5_ averages of 30.4 µg/m^3^ in 2022 [86]. Moreover, legacy pollutants such as dioxins (TCDD) and uranium in Vieques distinguish PR’s exposure profile from most Caribbean neighbors. Though overall cancer incidence in PR is slightly lower than in the mainland US, site-specific elevations exist [87,88]. Vieques, historically impacted by military training and munitions disposal, shows a 23–27% higher cancer incidence rate compared to the main island [89]. Biomonitoring in 2013 documented elevated arsenic, lead, and mercury levels among residents, suggesting legacy exposure [8]. Historical data also correlate intense bombing periods to reduced birth weights and preterm births [8]. This evidence reflects site-specific contamination from historic military activity rather than regional industrial trends, leading to a unique additional exposure risk. Taken together, PR exhibits a hybrid risk profile. Common pollutants like PM_2.5_ fall below US averages and neighbor Islands, while select HAPs such as EtO exceed mainland emissions, situating certain Puerto Rican communities in elevated or high carcinogenic risk categories under US EPA thresholds.

Collectively, modeled cancer risks, environmental measurements, and health indicators suggest that air toxics pose a serious but under-characterized risk to public health in PR. While existing studies highlight plausible exposure-disease associations in impacted communities, the absence of longitudinal epidemiological cohorts and integrated biomonitoring hinders causal attribution. Addressing these data gaps is essential to inform targeted interventions and environmental justice efforts on the island.

**Table 3:** Air pollutant concentrations in Puerto Rico (PR) with associated inhalation risk metrics (midpoint, IUR, RfC, LCR, CRL, HQ) and interpretations based on health impacts and regulatory thresholds.

Air Pollutants	Concentration (µg/m^3^) [7,9,10,27,44,63,90,91,92,93]	Years	Location	Midpoint (µg/m^3^)	IUR per (µg/m^3^) ^a^ [7,63,94,95,96,97,98]	RfC (mg/m^3^) ^b^ [7,63,94,95,96,97,98,99,100]	LCR ^c^	CRL ^d^ [101]	HQ ^e^
1,3-Butadiene	0.1–0.3	2015–2016	Salinas	0.2	3.00 × 10^−5^	2.00 × 10^−3^	6.00 × 10^−6^	Moderate	0.10
Acetaldehyde	1.0–2.0	2015–2016	Salinas	1.5	2.20 × 10^−6^	3.00 × 10^−5^	3.30 × 10^−6^	Moderate	50
Acrolein	0.02–0.05	2015–2016	Salinas	0.04		2.00 × 10^−5^			1.75
NH_3_	1.0–3.0	2015–2016	Salinas	2.0		0.1			2.00 × 10^−2^
Arsenic (Inorganic)	0.0005–0.0023	2015–2016	Salinas	0.0014	4.30 × 10^−3^	1.50 × 10^−5^	6.02 × 10^−6^	Moderate	9.33 × 10^−2^
Benzene	0.2–0.5	2015–2016	Salinas	0.35	7.80 × 10^−6^	3.00 × 10^−2^	2.73 × 10^−6^	Moderate	1.17 × 10^−2^
BaP (PAH)	0.0001–0.0005	2015–2016	Salinas	0.0003	6.00 × 10^−4^	2.00 × 10^−6^	1.80 × 10^−7^	Low	0.15
Cadmium	0.003–0.007	2015–2016	Salinas	0.005	1.80 × 10^−3^	1.00 × 10^−5^	9.00 × 10^−6^	Moderate	0.50
CO	600–10,000	2017	San Juan	5,300		23.0			0.23
Chloroform	0.1–0.3	2015–2016	Salinas	0.2	2.30 × 10^−5^	1.95 × 10^−3^	4.60 × 10^−6^	Moderate	0.10
Chromium VI	0.0001–0.0005	2015–2016	Salinas	0.0003	1.80 × 10^−2^	3.00 × 10^−5^	5.40 × 10^−6^	Moderate	1.00 × 10^−2^
Diesel PM	7.0–12.0	2017–2018	San Juan, Bayamón	9.5	3.00 × 10^−4^		2. 85 × 10^−3^	High	
Ethylbenzene	0.6–1.5	2015–2016	Salinas	1.05	2.50 × 10^−6^	1.0	2.63 × 10^−6^	Moderate	1.05 × 10^−3^
EtO	0.3–121	2023	Salinas	60.7	3.00 × 10^−3^	3.00 × 10^−5^	0.18	High	2,022
Formaldehyde	1.0–3.0	2015–2016	Salinas	2.0	1.30 × 10^−5^	8.00 × 10^−3^	2.20 × 10^−5^	Elevated	0.29
Lead (Inorganic)	0.05–0.2	2015–2016	Salinas	0.125	1.20 × 10^−5^		1.50 × 10^−6^	Moderate	
Mercury (Elemental)	0.0005–0.0015	2015–2016	Salinas	0.001	3.00 × 10^−4^	3.00 × 10^−4^	3.00 × 10^−7^	Low	3.33 × 10^−3^
Methylene Chloride	0.1–0.5	2015–2016	Salinas	0.3	1.00 × 10^−8^	0.60	3.00 × 10^−9^	Low	5.00 × 10^−4^
MTBE	0.5–1.0	2015–2016	Salinas	0.75	2.60 × 10^−7^	30.0	1.95× 10^−7^	Low	2.50 × 10^−5^
Nickel (dust)	0.0012–0.0034	2015–2016	Salinas	0.0023	2.60 × 10^−4^	1.00 × 10^−5^	5.52 × 10^−7^	Low	0.23
NO_2_	2.4–97	2016–2017	Peñuelas	49.7		0.47			0.11
O_3_	50–100	2015–2017	San Juan, Ponce	75.0		0.18			0.42
Phosgene	0.1–0.3	2015–2016	Salinas	0.2		1.00 × 10^−4^			2.0
PM_2.5_	1.4–45.9	2015–2016	Adjuntas, Ponce, Bayamón, Guayama, Fajardo, Guaynabo, Guayanilla, Humacao, San Juan	23.7					
Silica (crystalline, PM_10_)	0.3–0.6	2015–2016	San Juan	0.45		3.00 × 10^−3^			0.15
SO_2_	10–80	2017	San Juan, Guayama	45.0		2.62 × 10^−2^			1.72
Styrene	0.5–1.5	2015–2016	Salinas	1.0	7.00 × 10^−7^	1.00	7.00 × 10^−7^		1.00 × 10^−3^
TCDD	0.000005–0.00002		Peñuelas, Vieques	0.000013	38.00	4.00 × 10^−8^	4.75 × 10^−4^	High	0.31
TNT ^f^	0.2–0.4	Historical (2000s)	Vieques	0.3		0.0003			1.00
Toluene	0.5–1.5	2015–2016	Salinas	1.0		5.00			2.00 × 10^−4^
Uranium	0.1–0.3	2003–2005	Vieques	0.2		4.00 × 10^−5^			5.00
Vanadium (Pentoxide)	0.0005–0.0015	2015–2016	San Juan, Salinas	0.001	8.30 × 10^−3^	7.00 × 10^−6^	8.30 × 10^−6^	Moderate	0.14
Vinyl Chloride	0.1–0.5	2015–2016	Salinas	0.30	4.40 × 10^−6^	0.10	1.32 × 10^−6^	Moderate	3.00 × 10^−3^
Xylenes	1.0–1.5	2015–2016	Salinas	1.25		0.10			1.25 × 10^−2^

Notes: ^a^ IUR (Inhalation Unit Risk): Represents the estimated risk of cancer from inhaling a substance over a lifetime from EPA IRIS or OEHHA databases. ^b^ RfC (Reference Concentration): Indicates the daily inhalation exposure level for the human population that is likely to be without an appreciable risk of deleterious effects during a lifetime. ^c^ Lifetime Cancer Risk (LCR) = Concentration (µg/m^3^) × IUR (µg/m^3^)^−1^; LCR is interpreted based on standard risk categories. ^d^ Cancer Risk Level (CRL): ≥1 × 10^−4^: High risk; ≥1 × 10^−5^: Elevated risk; ≥1 × 10^−6^: Moderate risk; <1 × 10^−6^: Low risk; ^e^ Non-Cancer Risk (Hazard Quotient—HQ), HQ = Concentration (µg/m^3^) ÷ (RfC × 1000); HQ > 1 indicates possible concern for non-cancer effects. ^f^ The recommended airborne exposure limit TNT (REL) established by the National Institute for Occupational Safety and Health (NIOSH) used for RfC; Empty cells indicate that no authoritative IUR or RfC values are available from IRIS/OEHHA/ATSDR, or the pollutant is assessed using alternative health endpoints (e.g., blood lead levels, ambient air standards). Occupational exposure limits were not used. Further details are provided in Supplementary Material S1.

## 6. Mechanisms of Respiratory-Related Carcinogenesis by Air Pollutants

**Figure 2:**
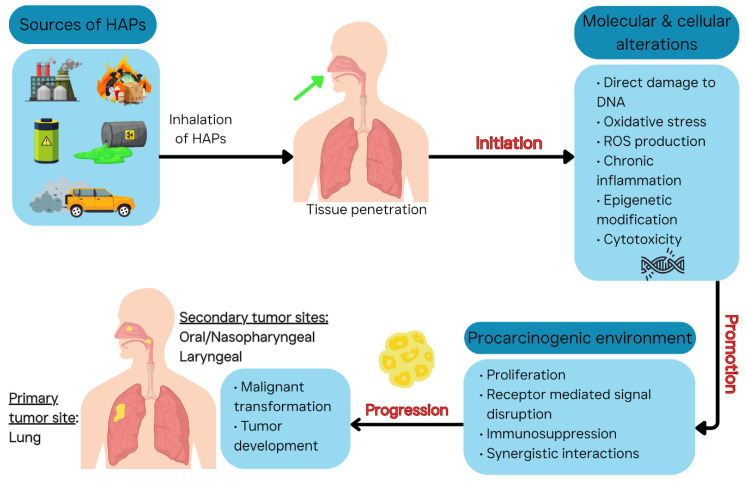
Multi-step carcinogenesis process developed by hazardous air pollutants (HAPs) exposure.

Growing evidence links air pollution to increased cancer incidence, underscoring the importance of clarifying the biological pathways that mediate this association. In this review, we focus on respiratory-related cancers, which represent the most commonly associated malignancies arising from exposure to air pollutants. HAPs can initiate and promote cancer through multiple biological pathways (Figure 2).

Carcinogenesis from inhaled pollutants is a multi-step process: initiation (inducing genetic damage in cells), promotion (fostering an environment where damaged cells proliferate), and progression (promoting malignant transformation and tumor growth). HAPs, depending on their chemical nature, may act at one or more stages by causing genetic instability, oxidative stress, chronic inflammation, epigenetic alterations, cytotoxicity/proliferation followed by compensatory proliferation, receptor-mediated signaling disruptions, immunosuppression, and synergistic interactions from mixed exposures. Table 4 summarizes key mechanisms and relevant air pollutants.

Many HAPs are genotoxic, meaning they directly interact with DNA, causing mutations in genes critical to cancer initiation [102]. Consequently, HAPs can induce TP53 mutations, which are commonly observed in cancers of the lung, larynx, nasopharynx, and oral cavity due to their direct exposure to inhaled toxins [103]. For example, PAHs like BaP are metabolized in the body to reactive intermediates that form DNA adducts—essentially pieces of PAH chemically bound to DNA [4]. EtO directly alkylates DNA bases (e.g., BRCA1/2), while benzene metabolites are associated to chromosomal breaks, linking exposure to leukemia (e.g., RUNX1) [63] and underscoring the hematologic impact of airborne hazardous exposures. In addition, EGFR amplifications are frequently found in lung cancers in response to environmental carcinogens (e.g., benzene, acrolein) [103]. Studies have found significantly higher mutation burdens in lung cancers from polluted environments compared to low-pollution areas, consistent with pollution-driven DNA damage [104].

**Table 4:** Molecular and cellular mechanisms of carcinogenesis by hazardous air pollutants (HAPs).

Mechanism	Description	Example HAPs [4,6,63]
Direct DNA Damage (Genotoxicity)	Chemical binds to or chemically alters DNA, causing mutations if not repaired.	PAHs (e.g., BaP) EtO Benzene metabolites
Oxidative Stress and ROS	Overproduction of reactive oxygen species leading to DNA strand breaks, base damage; also, lipid peroxidation.	Diesel PM and ultrafine particles Arsenic O_3_
Chronic Inflammation	Persistent activation of immune/inflammatory cells, releasing cytokines and ROS, promoting cell proliferation and DNA damage.	PM Wood smoke Endotoxin
Epigenetic Modification	Changes in gene expression without DNA mutation: DNA methylation, histone modification, microRNAs.	Traffic-related air pollution Nickel and arsenic Diesel exhaust
Cytotoxicity and Proliferation	Cell injury or death followed by regenerative proliferation increases risk of cancerous growth.	Formaldehyde Acrolein Strong acids or alkalis in aerosols
Receptor-Mediated Pathways	Activation of cellular receptors that drive proliferation or inhibit apoptosis.	Dioxins/PAHs Endocrine disruptors Pesticides Bisphenol A in dust
Immunosuppression	Impaired immune surveillance of tumors.	Dioxins (TCDD) and PCBs PAHs
Interaction of Mixed Exposures	Synergistic or additive effects of multiple pollutants.	Tobacco smoke + asbestos Diesel PM + viruses

Inhaled pollutants, especially particulates (e.g., diesel PM, coal ash dust), can trigger chronic inflammation in respiratory tissues, generating reactive oxygen species (ROS) and inflammatory cytokines [105]. Persistent ROS damage DNA, proteins, and cell membranes lipids, increasing genetic errors and promoting tumor development [106]. For instance, long-term exposure to fine PM is associated with chronic lung inflammation [107,108]; where animal studies have shown that particles can induce tumors in lungs partly via an inflammation-mediated pathway [109]. Metals (e.g., iron, copper, cadmium, mercury, nickel, lead, and arsenic) also generate ROS and impair antioxidant defenses, compounding oxidative DNA damage [110].

Beyond direct DNA mutations, air pollutants (PM_2.5_ and PM_10_) significantly impact epigenetic regulation, altering gene expression without modifying DNA sequences, known as DNA methylation (adding or removing methyl groups on cytosine bases in DNA [111,112,113]. Histone modifications (which affect how DNA is packaged) and microRNA expression changes have also been observed with pollutant exposure [114]. These epigenetic changes can be an early step in carcinogenesis and might serve as biomarkers of exposure.

Highly reactive pollutants like formaldehyde are associated with significant cytotoxicity, particularly to nasal epithelium cells, correlating to repeated regenerative proliferation cycles that increase spontaneous mutations and cancer risk [115].

Pollutants such as dioxins, dibenzofurans and non-ortho substituted PCBs and PAHs activate cellular receptors, like the aryl hydrocarbon receptor (AhR), influencing gene expression related to cellular proliferation and differentiation, potentially enhancing carcinogenesis [116]. EtO’s epidemiological link to breast cancer suggests additional hormonal pathways complementing its genotoxic effects [117,118].

Long-term exposure to certain HAPs (e.g., dioxins and heavy metals) can suppress aspects of the immune system, crucial for tumor surveillance. Reduced immune efficiency allows nascent tumor cells to evade immune elimination, thus facilitating tumor establishment and progression. Animal studies confirm increased tumor incidence due to immunotoxic correlations of pollutants like dioxins [63,119,120]. In reality, human exposure typically involves pollutant mixtures, resulting in interactions that enhance carcinogenic risk beyond individual associations. Metals such as arsenic and cadmium can inhibit DNA repair mechanisms, exacerbating genetic damage from co-exposures [121]. Diesel PMs facilitate deeper lung penetration of adsorbed carcinogenic PAHs, amplifying their potency [122,123]. Such interactions underscore the complex and compounded risks inherent in real-world exposures. Measures of dose–response evaluation make an effort to classify the connections between pollution exposure and subsequent health outcomes [124,125]. Data from past scientific investigations demonstrates that populations residing close to pollutant sources or deposition areas frequently suffer adverse health effects from unmanaged pollutant exposures contaminating air, water, soil and food supplies [126]. Despite methodological constraints and knowledge gaps, environmental health risk assessment methodologies incorporate exposure parameters such as pollutant concentration and duration, as well as socioeconomic factors to estimate health risks [127,128].

## 7. Other Respiratory Conditions Induced by Air Pollutants

Several HAPs have also been found to be associated with an array of adverse health outcomes, including respiratory and cardiovascular disease, and developmental disorders. Although randomized controlled trials are challenging, hence, rare due to ethical and practical limitations, substantial evidence from observational studies, case series, and toxicological research supports associations between HAP exposure and adverse health risks [129]. The US EPA and WHO have identified several HAPs benzene, and formaldehyde as significant contributors to exacerbation of chronic and respiratory diseases, impaired lung function and premature mortality [29,130]. Some HAPs, including isocyanates and formaldehyde, are also known as occupational asthmagens and may contribute to asthma initiation or worsening [131,132]. A correlation has been observed between specific complex mixes of HAPs, such as CO, NO_2_, SO_2_, O_3_, and PM_2.5_ and PM_10_, with an increased incidence of respiratory symptoms and hospital admissions due to asthma [133]. Correlative evidence links HAP exposure to increased hospitalizations for asthma and other respiratory conditions, though exact mechanisms and causality remain under investigation [129,134].

Emerging research suggests that fine PM, particularly from biomass burning or traffic emissions, penetrates deep into alveolar spaces and initiates inflammatory responses via activation of airway macrophages and epithelial cells [129]. These biological processes align with mechanistic models of chronic obstructive pulmonary disease (COPD), lung cancer and, potentially, other respiratory disorders like tuberculosis, and pneumoconiosis [106,135]. The complexity of pollutant mixtures and variable exposure patterns complicates attribution, yet mechanistic plausibility remains robust [129].

Although establishing definitive causal links between respiratory disorders and HAP exposure and respiratory disorders remains complex, robust observational and mechanistic evidence supports the carcinogenic classification of outdoor air pollution by agencies such as the IARC [62,136].

Understanding these mechanisms enables the identification of biomarkers for exposure and early problems, enabling targeted interventions such as antioxidants or policy-driven emission reductions. This mechanistic insight highlights the heightened risks within communities simultaneously exposed to multiple pollutants (e.g., PM and EtO) as observed in Puerto Rican populations. Biomonitoring studies conducted locally (e.g., PAH-DNA adduct levels comparing industrial versus rural areas) emphasize the need for expanded research on these mechanisms in affected populations.

## 8. Limitations and Recommendations

*Limitations of Current Knowledge*: While this review synthesizes the available evidence on air pollutants and their carcinogenic risks in PR, it is critical to acknowledge the inherent limitations of the existing research. A primary challenge is the sparse air quality monitoring network across the island, which has limited spatial coverage and may not capture localized pollution hotspots in industrial corridors or underserved communities. This lack of granular data has been observed alongside to a significant risk of exposure misclassification in health studies, potentially underestimating the true relationship between pollutants and health outcomes. Furthermore, many epidemiological studies conducted in PR are inherently *small-n* studies due to the island’s population size, which can limit statistical power and make it difficult to establish definitive causal links between specific pollutant exposures and cancer incidence.

*Policy and Public Health Recommendations*: Based on the findings of this review and in light of the identified limitations, we propose the following recommendations to protect public health and advance environmental health research in PR:

a. Prioritize the expansion of the island’s air monitoring network by installing new, real-time sensors in high-risk industrial and residential areas to fill critical data gaps.
b. Increase funding for large-scale, longitudinal cohort studies in PR to overcome the limitations of *small-n* studies.
c. Review and strengthen regulations on industrial emissions of known carcinogens and HAPs, and ensure robust enforcement to protect nearby communities.
d. Develop and maintain a publicly accessible platform or database that provides real-time air quality data and clear health advisories for all municipalities, empowering residents to take protective measures.

Implementing these measures is a crucial next step to translate research into evidence-based policies that can mitigate exposure to harmful air pollutants and safeguard the health of all residents in PR.

## 9. Conclusions

PR presents a compelling case study in the intersection of environmental health, policy, and environmental justice. This review highlights the influence of HAPs, including emissions from historical, military activities petrochemical facilities, vehicular traffic, natural environmental dust, coal combustion, and EtO sterilizers, on air quality and potential health outcomes. Modeled risk assessments and emerging epidemiological data suggest elevated cancer and respiratory disease risks in communities adjacent to major pollution sources, with disproportionately high burdens among low-income and marginalized populations.

Despite these concerns, critical knowledge gaps persist. Limited local monitoring, sparse longitudinal health data, and insufficient exposure assessment have hindered definitive causal inference. Nonetheless, the toxicological profiles of key pollutants, especially known carcinogens such as EtO, TCDD and Diesel PM warrant precautionary action. Regulatory efforts, such as the EPA’s updated EtO rules and PR’s coal phase-out, represent necessary, though overdue, progress.

To ensure the effectiveness of these interventions, their outcomes should be systematically assessed through longitudinal tracking of environmental pollutant levels and associated health metrics. Continued investment in monitoring infrastructure, enforcement mechanisms, and community-engaged research is essential to ensure accountability and sustained public health benefits. Under the Energy Public Policy Act (Act 17-2019), PR is mandated to achieve 100% renewable energy by 2050 [137]. Consequently, careful attention must be paid to secondary pollutants like O_3_, formaldehyde, and nitrogen oxides that may arise during this energy transition. While this transition is supported by US Department of Energy initiatives and integrated resource plans prioritizing solar and battery storage, interim reliance on natural gas raises concerns about methane leaks and combustion-related emissions that must be actively managed [137].

The Puerto Rican experience underscores how geographic isolation, colonial governance, and limited regulatory capacity can amplify environmental vulnerability. It also illustrates enduring environmental injustice, as exemplified by the long-standing contamination in Vieques, coal ash exposure in Guayama/Peñuelas, and elevated cancer risks in Salinas. Addressing these disparities requires coordinated action by scientists, public health practitioners, and policymakers.

Ultimately, advancing environmental health in PR will contribute to the broader understanding of air pollution impacts in tropical and island contexts, settings often underrepresented in the global literature. It also affirms the central aim of environmental public health: to safeguard all communities, especially those historically overlooked, from the harms of pollution. In particular, there is a pressing need for well-designed, large-scale studies evaluating the link between air pollution and cancer in Puerto Rican populations. Generating such evidence will be critical for informing policy, validating community concerns, and guiding long-term prevention strategies tailored to the island’s unique environmental and socio-political context.

## Data Availability

No new data were created or analyzed in this study. All data utilized are from publicly available sources, as cited throughout the text and reference list. The calculations presented in Table 3 LCR, CLR, and HQ) were derived from these existing datasets using standard risk assessment equations.

## Supplementary Materials

The following supporting information can be downloaded at: https://www.mdpi.com/article/10.3390/ijerph22101549/s1, Supplementary Material S1. Definitions and Sensitivity Analysis of Inhalation Risk Metrics Purpose [7,9,10,27,44,63,90,91,92,93,94,95,96,97,98,99,100,101,138]; Table S1: Air Pollutants sensitivity analysis of risk metrics.
