# Supplementary material for: Air Pollutants in Puerto Rico: Key Pollutants and Carcinogenic Properties
Source: Int J Environ Res Public Health. 2025 Oct 11;22(10):1549. doi: 10.3390/ijerph22101549 (PMC12564895; doi:10.3390/ijerph22101549)
Supplement: Supplementary file 1 [file ijerph-22-01549-s001.zip › ijerph-3856693-supplementary.pdf]

Supplementary Material S1. Definitions and Sensitivity Analysis of Inhalation Risk Metrics Purpose

This supplementary material provides quantitative uncertainty estimates for the inhalation-based cancer and non-cancer risk metrics reported in Table 3 of the main manuscript. The midpoint concentration, calculated as the arithmetic mean of this range, was used to derive the representative LCR and HQ values presented in **Table 3** in the main manuscript.

It is important to understand the nature of the toxicity values used in these calculations: **IURs** are upper-bound estimates designed to be health-protective; the actual risk is unlikely to exceed the calculated LCR values. **RfCs** incorporate multiple uncertainty factors (e.g., for interspecies and intraspecies differences) to derive a reference level that is protective of sensitive populations. All toxicity values were obtained from authoritative sources, primarily the EPA's Integrated Risk Information System (IRIS) and California's Office of Environmental Health Hazard Assessment (OEHHA).The following definitions and equations were used for the risk metrics presented in **Table 3** in main text:

Metric	Full Definition	Equation	Source for IUR/RfC Values
Inhalation Unit Risk (IUR)	Upper-bound estimate of lifetime cancer risk from continuous exposure to 1 µg/m³ over 70 years.	N/A (taken from IRIS/OEHHA databases)	[7,63,94-98,138]
Reference Concentration (RfC)	Continuous inhalation exposure level likely to be without appreciable non-cancer effects over a lifetime.	N/A (taken from IRIS/OEHHA)	[7,63,94-100]
Lifetime Cancer Risk (LCR)	The estimated upper-bound probability of developing cancer over a 70-year lifetime of continuous exposure to the measured air concentration.	LCR = C × IUR	Derived from IUR
Cancer Risk Level (CRL)	A qualitative interpretation of the quantitative LCR, categorizing the risk based on public health benchmarks (e.g., 1 in 10,000, 1 in 100,000, 1 in 1,000,000).	High ≥1E-4; Elevated ≥1E-5; Moderate ≥1E-6; Low <1E-6	[101]
Hazard Quotient (HQ)	The ratio of the estimated exposure (midpoint concentration) to the level at which no adverse non-cancer health effects are expected (RfC). An HQ>1 indicates that the exposure may warrant concern for potential non-cancer effects.	HQ = C ÷ [RfC × 1000] *	Derived metric

*Factor of 1000 converts RfC units from mg/m³ to µg/m³.

Midpoint Concentration Calculation: The midpoint concentration was chosen as the representative exposure value for the primary risk calculations in Table 3. This value was calculated as the arithmetic mean of the reported minimum and maximum concentration range for a given pollutant and location:

$$\text{Midpoint Concentration } (\mu g/m^3) = (\text{Minimum Concentration} + \text{Maximum Concentration}) / 2$$

For pollutants where only a single average value was reported in the original source material (e.g., Diesel PM, $PM_{2.5}$), that single value was used as the midpoint concentration. This approach is standard practice in screening-level risk assessments.

Calculated Risk Ranges (Sensitivity Analysis Results): We performed a sensitivity analysis by recalculating LCR and HQ at both the minimum and maximum reported concentrations for each pollutant. This provided LCR_{min}, LCR_{max} , HQ_{min}, and HQ_{max} applicable pollutants, are provided in **Table S1**.

Table S1. Air Pollutants sensitivity analysis of risk metrics.

**Air Pollutant**	**Conc. Range (μg/m^3) [7,9,10,27,44,90-93]**	**IUR per (μg/m^3) [**7,63,94-98**]**	**RfC (mg/m^3) [**7,63,94-98**]**	**LCR Range**	**HQ Range**
1,3-Butadiene	0.10 – 0.30	3.00×10^{-5}	2.00×10^{-3}	3.00×10^{-6} – 9.00×10^{-6}	5.00×10^{-2} – 1.50×10^{-1}
Acetaldehyde	1.00 – 2.00	2.20×10^{-6}	3.00×10^{-5}	2.20×10^{-6} – 4.40×10^{-6}	33.33 – 66.67
Acrolein	0.02 – 0.05		2.00×10^{-5}		1.0 – 2.5
Ammonia	1.00 – 3.00		0.10		1.00×10^{-2} – 3.00×10^{-2}
Arsenic (Inorganic)	0.0005 – 0.0023	4.30×10^{-3}	1.50×10^{-5}	2.15×10^{-6} – 9.89×10^{-6}	3.33×10^{-2} – 1.53×10^{-1}
Benzene	0.50 – 1.50	7.80×10^{-6}	3.00×10^{-2}	1.56×10^{-6} – 3.90×10^{-6}	1.67×10^{-2} – 5.00×10^{-2}
Benzo[a]pyrene (PAH)	0.0001 – 0.0005	6.00×10^{-4}	2.00×10^{-6}	6.00×10^{-8} – 3.00×10^{-7}	5.00×10^{-2} – 2.50×10^{-1}
Cadmium	0.003 – 0.007	1.80×10^{-3}	1.00×10^{-5}	5.40×10^{-6} – 1.26×10^{-5}	3.00×10^{-2} – 7.00×10^{-2}
Carbon Monoxide (CO)	600 – 10,000		23.00		2.61×10^{-2} – 4.35×10^{-1}
Chloroform	0.10 – 0.30	2.30×10^{-5}	1.95×10^{-3}	2.30×10^{-6} – 6.90×10^{-6}	5.13×10^{-2} – 1.54×10^{-1}
Chromium VI	0.0001 – 0.0005	1.80×10^{-2}	3.00×10^{-5}	1.80×10^{-6} – 9.00×10^{-6}	3.33×10^{-3} – 1.67×10^{-2}
Diesel PM	7.00 – 12.00	3.00×10^{-4}		2.10×10^{-6} – 3.60×10^{-3}	
Ethylbenzene	0.60 – 1.5	2.50×10^{-6}	1.00	1.50×10^{-6} – 3.75×10^{-6}	6.00×10^{-4} – 1.50×10^{-3}
EtO	0.3 – 121	3.00×10^{-3}	3.00×10^{-5}	9.00×10^{-4} – 3.63×10^{-1}	10.0 – 4,290
Formaldehyde	1.00 – 3.00	1.10×10^{-5}	8.00×10^{-3}	1.30×10^{-5} – 3.90×10^{-5}	1.25×10^{-1} – 3.75×10^{-1}
Lead (Inorganic)	0.05 – 0.20	1.20×10^{-5}		6.00×10^{-7} – 2.40×10^{-6}	
Mercury (Elemental)	0.0005 – 0.0015	3.00×10^{-4}	3.00×10^{-4}	1.50×10^{-7} – 4.50×10^{-7}	1.67×10^{-3} – 5.00×10^{-3}
Methylene Chloride	0.10 – 0.50	1.00×10^{-8}	6.00×10^{-1}	1.00×10^{-9} – 5.00×10^{-9}	1.67×10^{-4} – 8.33×10^{-4}
MTBE	0.50 – 1.00	2.60×10^{-7}	30.0	1.30×10^{-7} – 2.60×10^{-7}	1.67×10^{-5} – 3.33×10^{-5}
Nickel (dust)	0.0012 – 0.0034	2.60×10^{-4}	1.00×10^{-5}	2.88×10^{-7} – 8.16×10^{-7}	1.20×10^{-1} – 3.40×10^{-1}
Nitrogen Dioxide (NO_2)	2.40 – 97.00		0.47		5.11×10^{-3} – 2.06×10^{-1}
Ozone (O_3)	50 – 100		0.18		2.78×10^{-1} – 5.56×10^{-1}
Phosgene	0.10 – 0.30		1.00×10^{-4}		1.00 – 3.00
$PM_{2.5}$	1.40 – 45.90				

Silica (crystalline, PM_{10})	0.30 – 0.60		3.00×10^{-3}		0.10 – 0.20
SO_2	10 – 80		2.62×10^{-2}		0.38 – 3.05
Styrene	0.5 – 1.5	7.00×10^{-7}	1.00	3.50×10^{-7} – 1.05×10^{-6}	5.00×10^{-4} – 1.50×10^{-3}
TCDD (Dioxin, 2,3,7,8-Tetrachlorodibenzo-p-dioxin)	0.000005 – 0.00002	38.00	4.00×10^{-8}	1.90×10^{-4} – 7.60×10^{-4}	0.13 – 0.50
2,4,6-Trinitrotoluene (TNT)[e]	0.20 – 0.40		3.00×10^{-4}		0.67 – 1.33
Toluene	0.50 – 1.50		5.00		1.00×10^{-4} – 3.00×10^{-4}
Uranium	0.10 – 0.30		4.00×10^{-5}		2.50 – 7.50
Vanadium (Pentoxide)	0.0005 – 0.0015	8.30×10^{-3}	7.00×10^{-6}	4.15×10^{-6} – 1.25×10^{-5}	7.14×10^{-2} – 2.14×10^{-1}
Vinyl Chloride	0.10 – 0.50	4.40×10^{-6}	0.10	4.40×10^{-7} – 2.20×10^{-6}	1.00×10^{-3} – 5.00×10^{-3}
Xylenes	1.00 – 1.50		0.10		1.00×10^{-2} – 1.50×10^{-2}

Foundational Methodologies

These methodologies are detailed in foundational U.S. EPA guidance documents, specifically: A Review of the Reference Dose and Reference Concentration Processes, Guidelines for Carcinogen Risk Assessment, and the Risk Assessment Guidance for Superfund (RAGS) [EPA/540/1-89/002].
